# Screening of Plant UDP-Glycosyltransferases for Betanin Production in Yeast

**DOI:** 10.1007/s12010-024-05100-4

**Published:** 2025-01-02

**Authors:** Christiane Glitz, Jane Dannow Dyekjær, Dovydas Vaitkus, Mahsa Babaei, Ditte Hededam Welner, Irina Borodina

**Affiliations:** The Novo Nordisk Foundation Center for Biosustainability, Technical University of Denmark, Kemitorvet Building 220, 2800 Kgs. Lyngby, Denmark

**Keywords:** UDP-glycosyltransferase, Betanin, Natural food colours, Yeast cell factory, *S. cerevisiae*, In vivo enzyme screening

## Abstract

To cover the rising demand for natural food dyes, new sources and production methods are needed. Microbial fermentation of nature-identical colours, such as the red pigment betanin, has the potential to be a cost-efficient alternative to plant extraction. The last step of betanin production is catalysed by a UDP-glycosyltransferase (UGT). To find a high-performing UGT, we screened 27 UGTs from different plant species and tested their ability to produce betanin in vivo in *Saccharomyces cerevisiae*. We identified two new UGTs likely involved in the betanin synthesis in the plant they derive from: CqGT2 (UGT73A37) from *Chenopodium quinoa* and BgGT2 (UGT92X1) from *Bougainvillea glabra.* The betanin-producing UGTs were also tested in *Yarrowia lipolytica*, where CqGT2 was the best-performing glycosyltransferase for betanin production. While it has previously been shown that the UGTs can glycosylate either betanidin or cyclo-DOPA to ultimately form betanin, the molecular mechanism behind the preference for the acceptor molecule has not been elucidated. Therefore, we performed in silico structural analysis to characterise the betanin-producing UGTs further, particularly by looking into their binding mechanism. The docking model suggested that a smaller binding site found in some UGTs only allows glycosylation of cDOPA, while a wider binding site allows glycosylation of both cyclo-DOPA and betanidin.

**Supplementary Information:**

The online version contains supplementary material available at 10.1007/s12010-024-05100-4.

## Introduction

Food colours play a crucial role in the appeal and perception of food and beverages, and natural or synthetic dyes are commonly used in the food industry to achieve the desired colour and make the products more attractive and appetising [1]. In recent years, there has been a worldwide shift from the primary use of synthetic colours towards natural colours due to rising concerns about potential health risks associated with synthetic dyes [2, 3]. Betalains are a class of water-soluble natural colours that comprise the yellow-orange betaxanthins and the red-violet betacyanins. The red pigment betanin is the most prominent of the betalains, and it is commonly used in confectionery, beverages, meat replacement or dairy products under the name “Beetroot Red” (E162) [4]. The natural colourant is extracted from beetroots, but due to low pigment content (0.4%), seasonal dependence, and competition with food crops for resources and land, the production process is inefficient [5, 6]. Alternative sources and production methods are thus needed to cover the increasing demand for natural food colours. To address this, betanin production has been engineered in traditional crops such as *Nicotiana benthamiana*, *Solanum lycopersicum* (tomato) and *Oryza sativa japonica* (rice), enabling red beet pigment production of up to 270 mg betanin per 100 g fresh weight [7–10]. However, these plant-based betanin production systems face problems similar to extraction from beetroot as they also depend on arable land and the climate. The production of nature-identical colours by microbial fermentation is an alternative that circumvents some of the drawbacks of plant-based systems. To assess the feasibility, we have previously performed a life-cycle analysis and techno-economic assessment of heterologous betanin production with yeast [7]. We could show the superiority of biotechnological production over the traditional extraction process in both sustainability and economic aspects if sufficiently high titres and yields can be reached. Small-scale production of up to 30 mg/L betanin has been achieved in *Saccharomyces cerevisiae* cells that expressed the relevant biosynthetic enzymes for betanin production [11–13]. Furthermore, we recently showed that integration of the biosynthesis pathway into *Yarrowia lipolytica* and metabolic engineering of the strain enabled the production of > 1 g/L betanin in fed-batch fermentations [7].

**Fig. 1:**
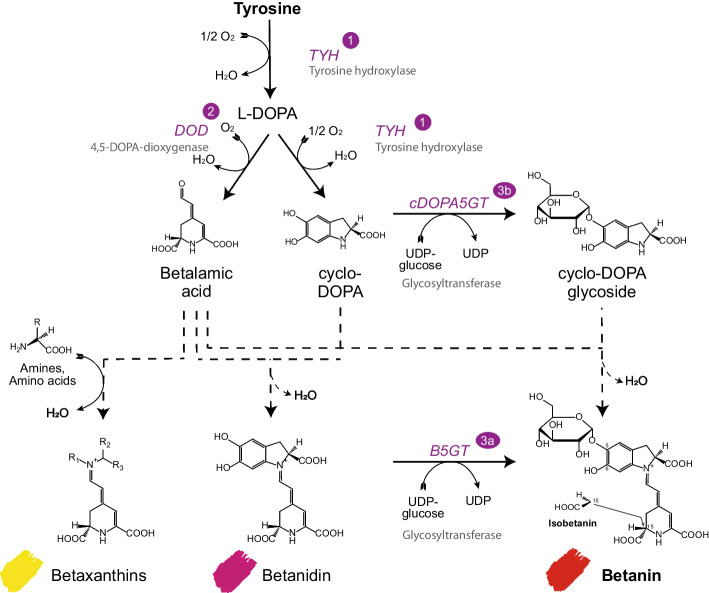
Simplified betalain biosynthesis pathway. Betanin is derived from tyrosine. Production of betanin in yeast requires three heterologous enzymes: a tyrosine hydroxylase (TYH), a DOPA-4,5-extradiol dioxygenase (DOD) and a UDP-glycosyltransferase (UGT) that is either active on cDOPA (cDOPA-5-O-glycosyltransferase - cDOPA5GT) or on betanidin (betanidin-5-O-glycosyltransferase - B5GT). Isobetanin is the C_15 _stereoisomer of betanin. Dashed arrows represent spontaneous reactions

Three enzymes are required to form betanin from L-tyrosine, the precursor of betalains (Fig. 1). First, L-tyrosine is oxidised to L-DOPA (3,4-dihydroxy-L-phenylalanine) by the monophenolase activity of a cytochrome P450 monooxygenase (1) of the CYP76AD family, herein tyrosine hydroxylase (TYH). Then, a DOPA-4,5-extradiol dioxygenase (DOD) (2) converts L-DOPA into 4,5-cyclo-DOPA which undergoes spontaneous cyclisation to become betalamic acid, the common chromophore of betalains. In parallel, L-DOPA is oxidised to dopaquinone by the TYH and then spontaneously cyclises to cyclo-DOPA (cDOPA). Betalamic acid can condense with primary and secondary amines to form betaxanthins, which are typically yellow and emit fluorescence. Betalamic acid can also undergo a spontaneous condensation reaction with cDOPA, forming the unstable violet-red intermediate betanidin (*λ*_max_ = ca. 540 nm) [14, 15]. In the last enzymatic step, betanidin is glycosylated at the C_5_-hydroxyl position by a regioselective UDP-glycosyltransferase (UGT) (3a), producing betanin (betanidin-5-O-β-glucoside). The attachment of the glucose leads to a slight hypsochromic shift (decrease in the absorption maximum wavelength) to 536 nm [16, 17]. Alternatively, cDOPA can first be glycosylated by a UGT (3b) to cDOPA-5-O-glucoside, which then condenses with betalamic acid to betanin [18]. As often observed for phenols, glycosylation drastically increases the stability of the compounds, mainly by impeding their oxidative degradation by blocking phenolic groups [19, 20]. Betanin is 17 times more stable than its aglycone when exposed to oxygen, and cDOPA is also expected to display higher oxidative stability when glycosylated [21].

Betalains are found in plants of the Caryophyllales order, e.g. *Beta vulgaris* (beetroot), *Chenopodium quinoa* (quinoa), *Bougainvillea glabra* (paper flower) or *Mirabilis jalapa* (four o'clock flower) to name a few [22]. To produce betanin heterologously, the three enzymes (TYH, DOD and UGT) involved in the biosynthesis of the pigment in plants are expressed in the respective production host. While multiple variants of TYHs and DODs have been screened to find the best combination for heterologous betanin production, only four UGTs have been reported to yield betanin heterologously so far: DbB5GT, MjcDOPA5GT, BvGT2, also known as UGT73A36 and BvGT4, also known as UGT73A39 [11–13, 23]. Glycosyltransferases are a diverse group of enzymes that catalyse the transfer of a sugar moiety to an acceptor molecule, resulting in the formation of a glycosidic bond [24]. In nature, many natural products, especially the secondary metabolites of microbes and plants, are glycosylated which changes the bioactivity and physio-chemical properties of the metabolites. The addition of a sugar moiety increases the polarity and solubility and often results in improved stability of the natural products [25]. The GTs involved in the glycosylation of natural products are mostly UDP-GTs, grouped into the GT-1 superfamily [26, 27]. In the case of UGTs, the sugar donor is an activated nucleotide diphosphate sugar (e.g. UDP-glucose) from which the sugar moiety is transferred with high stereo- and regiospecificity to the acceptor aglycone. The first UGTs involved in betanin biosynthesis were discovered in the 1990s in *Cleretum bellidiforme*, previously known as *Dorotheanthus bellidiformis* (DbB5GT and DbB6GT) [17, 28]. A few years later, Sasaki et al. described a UGT to be involved in betanin biosynthesis in *Mirabilis jalapa* (MjcDOPA5GT) and showed that this UGT transferred the sugar moiety to cDOPA instead of betanidin [18]. Babaei et al. reported two UGTs from *Beta vulgaris* that led to the production of betanin in an *S. cerevisiae* strain that expressed a TYH (BvTYH^W13L^) and a DOD (MjDOD) [12]. The highest betanin titre (30 mg/L) was reached with BvGT2. In recent years, the betanin biosynthesis of many plants has been investigated, and the responsible genes identified, including UGTs from dragon fruit (*Hylocereus* spp.), amaranth (*Amaranthus* spp.) or quinoa (*Chenopodium quinoa*) while in other plants, the enzymes responsible for betanin formation remain unknown [29–31].

In this study, we screened 27 UGTs from various plant species to identify additional enzymes that are efficient in glycosylating betanidin or cDOPA in the heterologous yeast system. The UGTs were selected from the NCBI database and from the transcriptome of *B. glabra*, which was assembled and annotated as part of this work. The 27 UGTs were first tested for betanin production in *S. cerevisiae*. Those enzymes that proved to be functional in the first screening were afterwards tested in *Y. lipolytica*. Furthermore, we performed a structural analysis and functional characterisation of the most promising UGTs, providing insights for optimising betanin production strains and mitigating product degradation.

## Materials and Methods

### Bioinformatic Selection of Plant UGTs

The glycosyltransferase BvGT2 from *Beta vulgaris* can sufficiently glycosylate betanidin to produce betanin in yeast [12]. This UGT was blasted against RefSeq plant (taxid:3193) sequences in NCBI databases, and we obtained 100 unique initial hit sequences. To limit the number of genes to a manageable amount, only the hits belonging to the Chenopodiaceae family, a subfamily of the Caryophyllales order that comprises the betalain-producing plants *B. vulgaris, Spinacia oleracea* and *C. quinoa,* were retained. For sequences belonging to families not previously known to form betalains, only one sequence with the highest identity to BvGT2 was retained per organism. By visual inspection of a multiple sequence alignment, the hits were checked for the presence of the UGT signature prosite pattern PS00375. Finally, we calculated the similarity/identity within the hit sequences using MatGat2.01 [32], and we removed six sequences that were 95% or above identical to other sequences, leaving 24 sequences for testing (Table 1).

### Bioinformatic Selection of UGTs from *Bougainvillea glabra*

We downloaded single-end Illumina HiSeq 2500 transcriptomic RNA-seq raw reads of red *Bougainvillea glabra* cultivar Sanderiana from NCBI (SRR1994279) and generated a fastq file using NCBI’s SRAToolKits.2.10.4 fastq-dump tool. We used FastQC [34] to do quality control and check the reads for overrepresented sequences. The reads were trimmed with TrimGalore-0.6.5 using a phred33 quality score [35]. The trimmed, unpaired fastq reads were de novo assembled using Trinity v2.9.1 [36]. To avoid memory shortage during the transcriptome assembly, we used the parameters –min_kmer_cov 2 and –normalize_reads. Calculating the assembly statistics with the TrinityStats.pl script in Trinity v2.9.1 resulted in a median transcript contig length of 331 with an N50 value of 474. Next, we calculated the expression levels with align_and_estimate_abundance.pl in Trinity v2.9.1 using bowtie as the alignment method and RSEM as the abundance estimation method. Finally, the longest open reading frames (ORFs) were extracted using TransDecoder [37]. Then, we annotated the transcriptome by blasting the transcripts and ORFs into local Uniprot databases. In addition, we extracted the PFAM domains for the ORFs. The results were loaded into an SQLite database using Trinotate v3.2.0 [38]. Finally, we merged the expression levels into a final annotation table. We searched the annotation table for UGTs by extracting sequences annotated as “scopoletin glucosyltransferase” and sequences having PF00201 motif (UDP-glucuronosyl- and UDP-glucosyltransferases). We blasted the transcriptome for the literature sequences CAB56231.1, AAL57240.1, AAS94330.1, AAS94329.1, BAD91803.1 and BAD91804.1, and the previously discovered sequences of BvGT1-4 (Supplementary File 2). To ensure that the transcripts start with AGT, all transcripts were subjected to the ORF finder module of the Sequence Manipulation Suite [39]. All protein sequences shorter than 400 amino acids were removed, and the remaining set was checked for the prosite pattern PS00375. Finally, sequences with more than 95% similarity to the remaining sequence were removed, resulting in 13 sequences. Of those, we selected three highly expressed sequences for testing.

### Phylogenetic Tree of UGTs and MSA

A multiple sequence alignment (MSA) of the UGTs was created with Clustal Omega (version 1.2.4) (EMBL-EBI) [40]. From that, the neighbour-joining phylogenetic tree data and the sequence identity matrix were obtained. The phylogenetic tree was displayed and edited with iTOL [41].

### Synthetic Genes and Oligonucleotides

Heterologous genes were synthesised as synthetic gene strings and codon-optimized for *S. cerevisiae* or *Y. lipolytica* by GeneArt (Thermo Fisher Scientific, US) or Twist Bioscience (USA). The corresponding amino acid and nucleotide sequences can be found in Supplementary File 2. Oligonucleotides were obtained from Integrated DNA Technologies, and a list of all oligonucleotides used in this work can be found in Supplementary File 2.

### Media and Cultivations

*Escherichia coli* DH5α strains, used for cloning and plasmid propagation, were cultivated in lysogeny broth (LB), supplemented with 100 mg/L ampicillin, at 37 °C, 200 rpm. During strain construction and when growing strains from glycerol stocks, *S. cerevisiae* strains were grown in yeast peptone dextrose media (YPD), supplemented with 200 mg/L Geneticin (G418) for Cas9-plasmid selection, *Y. lipolytica* strains were grown without G418. After transformation, yeast strains were plated on synthetic complete (SC) agar plates prepared with 76 mg/L of the standard amino acids, 380 mg/L leucine, 18 mg/L adenine, 7 mg/L inositol and 8 mg/L *p*-aminobenzoic acid (*p*ABA). For small-scale cultivations in 24-well plates, mineral media (MM), buffered with potassium phosphate (KH_2_PO_4_), was prepared with 7.5 g/L (NH_4_)_2_SO_4_, 14.4 g/L KH_2_PO_4_, 0.5 g/L MgSO_4_ 7**·**H_2_O, trace metals and vitamins, 20 g/L glucose and adjusted to pH 6.0 with NaOH [42]. The yeast strains were cultivated at 30 °C, 250 rpm. For agar plates, 20 g/L agar was added to the respective media.

### Plasmid and Strain Construction

*S. cerevisiae* strains were constructed according to the EasyClone MarkerFree toolkit, *Y. lipolytica* strains were constructed according to the EasyClone YALI toolkit [43, 44]. Promoters and heterologous genes were amplified by PCR with USER-compatible overhangs using Phusion U Hot Start DNA Polymerase (Thermo Fisher Scientific, USA) and assembled with PCR-linearised integration vectors in *E. coli* DH5α by USER Cloning [45]. The plasmids were purified from *E. coli* using a NucleoSpin plasmid miniprep kit (Macherey Nagel, Germany), and correct plasmid assembly was verified by Sanger sequencing (Eurofins Genomics, LU). Plasmids were linearised with FastDigest NotI (Thermo Fisher Scientific), resulting in a fragment containing the promoter, gene of interest, terminator and 500–600 bp upstream and downstream homology regions that navigate the integration site in the host genome. The integration fragment and the gRNA were transformed into Cas9-expressing *S. cerevisiae* or *Y. lipolytica* strains with the LiAc method [46]. Transformants were selected on SC plates containing 100 mg/L nourseothricin (*Y. lipolytica*: 250 mg/L nourseothricin) and 200 mg/L G418 (*S. cerevisiae* strains only). Correct integration of the fragment was verified by colony PCR using RedTaq MasterMix (VWR life science).

The *S. cerevisiae* strains used in this study were derived from the haploid strain CEN.PK113-7D (MATa *URA3 HIS3 LEU2 TRP1 MAL2-8c SUC2*). All *Y. lipolytica* strains generated in this study were derived from a W29/CLIB89 (NRRL Y-63746) strain containing a Cas9 expression cassette in the KU70 locus. Glycerol stocks of the created *E. coli* and yeast strains were made by adding glycerol to overnight cultures (25% (v/v)) and storing them at − 70 °C. A list of all biobricks, plasmids and strains created for this work can be found in Supplementary File 2.

### Small-Scale Cultivation in 24- and 96-Well Plates

Screening of the glycosyltransferases in *S. cerevisiae* was performed in 24-deep-well plates in 2 mL MM (without *p*ABA). The media was inoculated in a ratio of 1:50 (v/v) from a preculture grown for 16 h in MM (+*p*ABA), corresponding to a starting OD_660_ of ca. 0.1. After 48 h, the cultivation was stopped, and the cultures were analysed for cell growth (OD_660_), UV-Vis spectra, betaxanthin fluorescence and extracellular and total betanin content. The OD_660_ was measured in a plate reader using a previously determined correction factor between measurements in a cuvette (*l* = 1 cm) and the plate reader. To determine the betanin content in the supernatant of the yeast cultures, 1 mL of cultivation broth was centrifuged at 5000 x g for 10 min, the supernatant was transferred to 1.5-mL reaction tubes, centrifuged again, and finally, the supernatant was transferred to amber-coloured vials for HPLC analysis. To quantify total betanin content (intracellular + extracellular), 1 mL of cell culture was transferred into a 2-mL microtube (Sarstedt) containing ca. 0.25 mL of 0.5–0.75 mm glass beads. The cells were then disrupted using a Precellys R 24 homogeniser (Bertin Corp.) in five cycles of 5000 rpm for 30 s, with a cooling step on ice between each lysis cycle. After disruption, the tubes were centrifuged for 10 min at 10,000 g. The supernatant was transferred to 1.5-mL reaction tubes and centrifuged again before being transferred to amber-coloured vials for HPLC analysis. Samples were stored at −80°C after cell removal and extraction.

After transformation of the UGTs into the betanidin-producing platform strain, a first screening of betanin production was done by assessing the red colour the strains had secreted into the agar plates. The strains expressing UGTs that were able to make betanin could be differentiated by the reddish colouring of the agar plate. Those five strains (ST12160, ST12168, ST12170, ST12441 and ST12446) and the two control strains without integrated UGT (ST9942 and ST7574) were cultivated in triplicates in small-scale cultivation, whereas only one replicate of the other strains was cultivated. The titres produced by the strains were not normalised to the OD_660_ of the cultures because despite measuring the OD at 660 nm instead of 600 nm, there is still overlap with the maximal absorbance of the betacyanins (540 nm), which is likely to cause significant deviations in the measured OD values. The same protocol was followed to test the UGTs in *Y. lipolytica*. All five strains, ST12055 and ST14640–ST14643, were cultivated in biological triplicates.

The influence of different media compositions on the UGTs was investigated by cultivation in 96-well plates. The precultures were grown in 24-well plates, as described above. The main cultures were grown in 96-well plates with 500 μL of the respective media, inoculated to OD_660_ 0.2, for 48 h. The betanin and isobetanin production was quantified by measuring the absorbance of the supernatant at 535 nm in a plate reader. A previously determined correction factor (×14) was used to convert the absorbance into betalain concentration in mg/L.

### Expression Analysis of the UGTs in *S. cerevisiae*

To test whether the UGTs were expressed in the yeast cells, all betanin-producing UGTs and some non-producing UGTs were fused to a C-terminal 6xHis-tag (pCfB11832, pCfB11835-45, pCfB11887) and integrated into strain ST9942 using the cloning methods described above. YPD (25 mL) media were inoculated with the constructed strains to an OD_600_ of 0.1 from an overnight culture and grown in shake flasks for 7 h when an OD_600_ of ≈3 was reached. The cells were washed in 5 mL sodium phosphate buffer (50 mM NaPO_4_, pH 7.4), centrifuged again, and the cell pellet was stored at −20 °C. The next day, the cells were thawed and resuspended in 250 μL of CelLytic Y cell lysis reagent (Sigma-Aldrich, Germany). Protease inhibitor cocktail (Sigma-Aldrich) and 2.5 μL dithiothreitol (DTT) were added, and the cells were kept on ice for 15 min. The cells were additionally lysed with a Precellys R 24 homogeniser to increase the protein yield. The cell lysates were transferred into 2-mL microtubes (Sarstedt) containing ca. 0.25 mL of 0.5–0.75 mm glass beads and disrupted in three cycles of 6500 rpm. After disruption, the tubes were centrifuged for 5 min at 10,000 g. The supernatant was transferred to 1.5-mL reaction tubes and centrifuged again. The resulting supernatant (ca. 200 μL) contained the soluble proteins. 100 μL of lysis buffer (50 mM Tris-HCl pH 7.5; 100 mM NaCl) was added to the pellet in the microtubes containing the insoluble cell material and transferred to a 1.5-mL reaction tube. After another centrifugation step, the pellet was resuspended in 150 μL lysis buffer as insoluble protein fraction. The soluble and insoluble protein fractions were stored at −20 °C. The protein concentration was determined via bicinchoninic acid (BCA) assay (Thermo Fisher Scientific) according to the manufacturer’s instructions. This information was used to load the same amount of protein for sodium dodecylsulfate polyacrylamide gel electrophoresis (SDS-PAGE). The samples were incubated for 10 min at 95 °C in Sample Buffer (2×) (Thermo Fisher Scientific) with 30 mg/mL DTT. Mini-PROTEAN TGX Gels (Bio-Rad) were loaded with the samples and were run with a pre-stained protein ladder (Thermo Fisher Scientific) at 20 mA per gel for ca. 1.5 h. If no immunoblot was performed, the SDS gel was stained with InstantBlue Coomassie stain (Abcam, UK). For immunoblotting, the proteins were transferred via dry blotting onto an iBlot2 membrane (Invitrogen) and prepared according to the instructions of the Penta⋅ His HRP Conjugate kit (Qiagen). The membranes were incubated with SuperSignal West Pico Plus chemiluminescent substrate (ThermoFisher) for 5 min and analysed in a gel imager.

### Analytical Methods

The cultivations’ extracellular and total betanin and isobetanin contents of the cultivations were analysed via high-performance liquid chromatography (HPLC) using a Dionex Ultimate 3000 HPLC system (Thermo Fisher Scientific, US). The samples were run on a Zorbax Eclipse Plus C18 reverse-phased column (particle size 3.5 μm; pore size 95 Å; 4.6 × 100 mm). The column oven temperature was set to 30 °C and the flow rate to 1 mL/min, with 10 μL of sample injection. Solvent A was 0.1% formic acid, and solvent B was 100% acetonitrile. The solvent composition was initially *A* = 98.0% and *B* = 2.0% for 2 min. A linear gradient was run until *A* = 90.0% and *B* = 10.0% at 5.0 min. A second gradient was run until *A* = 85.0% and *B* = 15.0% at 8.0 min. At 8.2 min, A was set to 2.0% and B to 98.0%. This condition was kept constant until 9.5 min, after which the initial composition was retrieved (*A* = 98.0%; *B* = 2.0%) and remained unchanged until the end of the run (11.5 min). Betanin and isobetanin were detected with a UV-Vis detector at a wavelength of 540 nm at retention times of ca. 5.7 min and 6.1 min, respectively. The Chromeleon 7 software (Thermo Fisher Scientific, US) was used to analyse the HPLC results. As pure betanin standard is not commercially available, peaks corresponding to betanin and isobetanin were identified using red beet extract diluted with dextrin as standard (product ID:901266-5G). When analysing this sample, we observed an equimolar mixture of betanin and its C_15_-stereoisomer isobetanin. By using the Beer-Lambert equation, assuming a molar extinction coefficient of *ε* = 6.5 × 10^4^ M^−1^ cm^−1^ for both betanin and isobetanin [47], we determined that 1 g/L of this solution contains 0.84 mg/L of betanin and isobetanin. This data was used to create calibration curves for HPLC quantification.

Betaxanthins were quantified by measuring the fluorescence (excitation 463 nm; emission 512 nm) of the extracellular and total extracts of the cultivation in a plate reader (BioTek Elx 8089, USA). The optical density (OD) of the yeast cultures was measured at 660 nm (instead of 600 nm) to minimise the overlap with the max. absorbance of betanin at 535 nm. The UV–Vis spectrum of the total samples (extra- + intracellular) was measured with a plate reader (BioTek Elx 8089, US) from 300 to 700 nm in 5-nm steps.

### Protein Structure Modelling, Structure Analysis and Docking

Protein structural models were generated with AlphaFold v2.0, using all available structural homologs, and the database search preset was set to “reduced_dbs” [48]. Only the highest ranking (in pLDDT score) models were used in further analyses. Binary complexes of protein and sugar donor were obtained by structurally aligning protein model structures on the crystal structure of PtUGT1 from *Polygonum tinctorium*, which has a bound UDP-glucose molecule in its active site (6SU6.pdb) [49]. 6SU6 was chosen as template for the structure comparison because it has already been used in different studies that employed computational methods, including docking, molecular dynamics and QM-MM where reaction mechanisms for different glycosylations were established [49, 50]. Thus, it was deemed as a suitable structure to do the comparisons with. The substrates were added by docking into the acceptor binding site of the binary complexes, using gnina v1.0.1 software [51] a fork of smina [52], itself a fork of AutoDock Vina [53]. PyMOL (v2.4.0) was used to superimpose and visualise the resulting structures [54]. We used three parameters to define a reactive docking pose: (1) nucleophilic attack distance (distance C1_glc_-OH6′_sub_, threshold < 5 Å), deprotonation of the phenolic oxygen by the catalytic histidine (distance N_His_-OH6′_sub_, threshold < 3.5 Å), and orbital angles (angle between O1_glc-_C1_glc_-OH6’_sub_ and threshold > 130°).

## Results

### Selection of UGTs for Betanin Production

We have previously identified a UGT in beetroot (BvGT2), whose heterologous expression, together with BvTYH^W13L^ and MjDOD, resulted in the highest betanin titre reached in *S. cerevisiae* so far [12]. To find more UGTs that can glycosylate betanidin or cDOPA to make betanin, BvGT2 was blasted against RefSeq plant (taxid:3193) sequences in NCBI databases, and the best hits from plants of the Caryophyllales order as well as from plants of other orders were selected. The final hitlist comprised 24 UGTs, of which 14 originated from betanin-producing plants and ten from plants that do not produce betalains (Table 1). We included UGTs from plants that do not produce betalains because we were curious whether these UGTs could, in principle, make betanin but lack the appropriate precursors in their plant of origin. For the UGTs from betanin-producing plants, the final list comprised multiple genes from the same three species: *Chenopodium quinoa* (*CqGT1-6*), *Spinacia oleracea* (SoGT1-7) and one hit from *Beta vulgaris* (BvGT5). Most of these genes belonged to the UGT73 family. Since we did not use expression data that would verify the involvement of the selected genes in betalain biosynthesis, we decided to test multiple UGTs from the same organism. In addition, we were interested in glycosyltransferases from the betalain-making plant *Bougainvillea glabra*. Since no annotated genes of that plant were available in the databases, we assembled and annotated the transcriptome of *Bougainvillea glabra* cultivar Sanderiana and searched for UGTs as performed for the sequences in NCBI databases. Three UGTs (BgGT1-3) were selected from the hitlist based on their expression levels and annotations. In total, 27 UGTs were selected for in vivo testing in *S. cerevisiae*. The sequence identity matrix of the UGTs can be found in Supplementary File 3.

**Table 1:** List of UGTs screened in vivo for betanin production in *S. cerevisiae*. 27 UGT candidates were identified in the transcriptome of different plant species. In addition, DbB5GT*,* MjcDOPA5GT and BvGT2, previously shown to produce betanin in *S. cerevisiae* strains, were used as benchmarks. Plants labelled with an asterisk (*) produce betalains. Systematic UGT names after Mackenzie et al. [33]. Strain ID refers to *S. cerevisiae* strains

UGT	Systematic name	Origin	Strain ID	Protein ID (NCBI)
DbB5GT	UGT73A5	Dorotheanthus bellidiformis*	ST12158	CAB56231.1
MjcDOPA5GT	UGT92X2	Mirabilis jalapa*	ST12441	AZC85901.1
BvGT2	UGT73A36	Beta vulgaris*	ST12150	XP_010691725.1
BvGT5	UGT73A40	Beta vulgaris*	ST12153	XP_010691724.1
CiClGT1	UGT73A53	*Citrus clementina*	ST12303	XP_006422969.1
CiSiGT1	UGT73A54	*Citrus sinensis*	ST12302	XP_006487031.1
CpGT1	UGT73A55	*Carica papaya*	ST12305	XP_021887294.1
CqGT1	UGT73DN2	Chenopodium quinoa*	ST12154	XP_021718283.1
CqGT2	UGT73A37	Chenopodium quinoa*	ST12290	XP_021753346.1
CqGT3	UGT73A48	Chenopodium quinoa*	ST12291	XP_021753343.1
CqGT4	UGT73A49	Chenopodium quinoa*	ST12292	XP_021753345.1
CqGT5	UGT73A50	Chenopodium quinoa*	ST12293	XP_021753348.1
CqGT6	UGT73A51	Chenopodium quinoa*	ST12294	XP_021714530.1
CsGT1	UGT73DN2	*Camellia sinensis*	ST12301	XP_028053269.1
EgGT1	UGT73A60	*Eucalyptus grandis*	ST12304	XP_039172473.1
MeGT1	UGT73A57	*Manihot esculenta*	ST12306	XP_021624601.1
PaGT1	UGT73B45	*Prunus avium*	ST12155	XP_021823916.1
RaGT1	UGT73A61	*Rhodamnia argentea*	ST12307	XP_030525034.1
SoGT1	UGT73A41	Spinacia oleracea*	ST12156	XP_021851291.1
SoGT2	UGT73A42	Spinacia oleracea*	ST12295	XP_021851289.1
SoGT3	UGT73A43	Spinacia oleracea*	ST12296	XP_021851290.1
SoGT4	UGT73A44	Spinacia oleracea*	ST12297	XP_021851293.1
SoGT5	UGT73A45	Spinacia oleracea*	ST12298	XP_021839484.1
SoGT6	UGT73A46	Spinacia oleracea*	ST12299	XP_021863511.1
SoGT7	UGT73A47	Spinacia oleracea*	ST12300	XP_021851288.1
TcGT1	UGT73A63	*Theobroma cacao*	ST12308	XP_007042481.2
VrGT1	UGT73A62	*Vitis riparia*	ST12157	XP_034682237.1
BgGT1	UGT84A91	Bougainvillea glabra*	ST12173	
BgGT2	UGT92X1	Bougainvillea glabra*	ST12174	
BgGT3	UGT73DM1	Bougainvillea glabra*	ST12175	

### In Vivo Screening of UGTs in *S. cerevisiae*

**Fig. 2:**
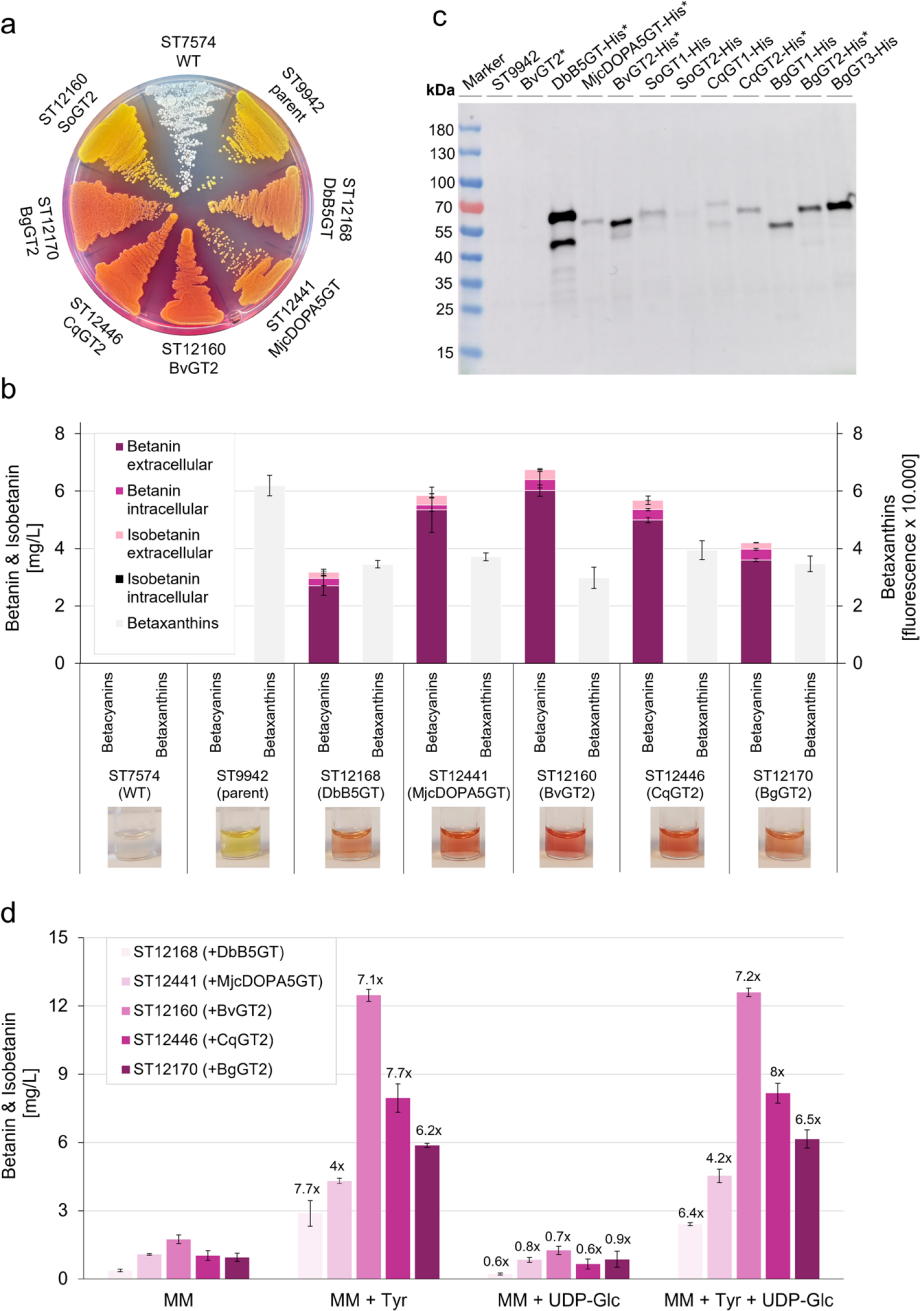
Betanin production in *S. cerevisiae* strains expressing different plant UGTs. **a** ST7574 (no betalain pathway genes), ST9942 (↑MjDOD, ↑BvTYH^W13L^) and strains expressing different UGTs on SC agar.** b** 27 UGTs were screened for their ability to produce betanin when integrated into the betaxanthin-producing yeast strain ST9942. The strains were cultivated in MM in 24-well plates. Betanin and isobetanin concentrations in the supernatant and total cell extract (supernatant + intracellular) were quantified by HPLC, betaxanthin production was assessed by measuring the betaxanthin-typical fluorescence profile of the total cell extract. Except for the control strains ST7574 and ST9942, only the strains that produced betanin are shown. The raw data for all tested strains can be found in Supplementary File 1, Table A1. The mean values of biological triplicates (± SDs) are shown. **c** Expression analysis of the UGTs by immunoblotting. All betanin-producing UGTs, marked with an asterisk (*), and a subset of non-producing UGTs were fused to a His-tag and integrated into strain ST9942. After cultivating the strains in YPD media, the cells were lysed, and the protein concentration of the soluble lysate fractions was determined by BCA. Total soluble protein (50 μg) was separated by SDS-PAGE and afterwards immunoblotted using an Anti-His HRP-conjugate and chemiluminescent detection. Controls: ST9942 (no UGT), BvGT2 (UGT without His-tag). Size of UGTs: ≈ 55 kDa. **d** Betanin production with different media compositions. The strains expressing the indicated UGT were cultivated in MM with a. no supplementation b. 500 mg/L tyrosine c. 1.6 mM UDP-glucose d. 500 mg/L tyrosine + 1.6 mM UDP-glucose for 48 h in 96-well plates. Betanin concentration was determined by measuring the absorbance of the supernatant at 535 nm and then converted to mg/L using a conversion factor. The supplementation with tyrosine drastically increased betanin production in all strains whereas addition of UDP-glucose led to a small decrease in betanin production. The combination of tyrosine and UDP-glucose supplementation had the same effect as only tyrosine supplementation. The fold-change between betanin production in MM without and with supplementation is indicated. Cultivation in 96-well plates decreased the betanin titres compared to cultivation in 24-well plates. The mean values of *n* = 4 replicates (± SDs) are shown

The 27 UGTs were tested for their capacity to produce betanin by integrating the codon-optimised genes in *S. cerevisiae* strain ST9942. This platform strain expressed the betalain pathway genes BvTYH^W13L^ and MjDOD and can produce the betanin precursors betanidin, betalamic acid and cDOPA [11, 12]. The resulting yeast strains are listed in Supplementary File 2. Strains expressing the UGTs DbB5GT, MjcDOPA5GT or BvGT2 were also constructed to benchmark the activity of the screened UGTs, as all three have previously been shown to produce betanin in betaxanthin-producing yeast. A first assessment of the UGTs’ capacity to produce betanin was done by eye after the transformation. After 3 days of incubation, strains expressing DbB5GT, MjcDOPA5GT, BvGT2, CqGT2 and BgGT2 had coloured the agar plate red, while all other strains, like ST9942, were bright yellow and turned the agar yellow-orange (Fig. 2a). All strains were cultivated in 24-well plates in MM. Afterwards, the OD_660_ was measured, and the total and extracellular betacyanin contents were analysed by HPLC. In addition, the UV-Vis spectrum (Supplementary File 1, Fig. A1) and betaxanthin fluorescence (excitation 463 nm; emission 512 nm) of the total cell extract were determined. As expected, a higher concentration of the yellow betaxanthins was found in strains not producing betanin than in betanin-producing strains. The HPLC analysis showed that none of the UGTs derived from plants that cannot produce betalains, produced betanin in the yeast. Of the newly screened UGTs, only the strains with integrated CqGT2 (ST12446) or BgGT2 (ST12170) produced betanin and isobetanin (Fig. 2b). Both strains produced more than the strain with integrated DbB5GT (ST12168), and CqGT2 was almost as efficient as MjcDOPA5GT in glycosylating the betanin precursor, resulting in ≈ 6 mg/L betacyanin. However, none of the UGTs produced as much as the strain expressing BvGT2 (total betacyanins 6.8 mg/L; betanin 6.4 mg/L betanin; isobetanin 0.35 mg/L). Isobetanin exhibits identical chromatic properties to betanin and is therefore considered a desired product in this work. In contrast to Beetroot Red (E162), where isobetanin makes up for up to 45% of the betacyanin content [4], all here tested yeast strains produced max. 7% isobetanin, while betanin accounted for over 90% of the total betacyanin concentration. In accordance with what we have reported previously, most of the produced betacyanins were secreted by the *S. cerevisiae* cells into the cultivation media, and only 3–10% were retained within the cells [12]. According to these results, the efficiency of the UGTs to produce betanin in this strain background was as follows: DbB5GT < BgGT2 < CqGT2 < MjcDOPA5GT < BvGT2. The HPLC chromatograms for all betanin-producing strains are shown in Supplementary File 1, Fig. A2, the raw data for the cultivated strains can be found in Table A1.

Interestingly, only two of the 27 screened UGTs, CqGT2 and BgGT2, could glycosylate the aglycone of betanin in the betaxanthin-producing yeast strain ST9942. To elucidate whether the other 25 UGTs did not produce betanin because they were not active on the betanin precursors or simply because they were not expressed in the yeast cells, we analysed the expression levels of a subset of UGTs, namely the betanin-producing DbB5GT, MjcDOPA5GT, BvGT2, CqGT2, BgGT2 and of five non-producing UGTs (SoGT1, SoGT2, CqGT1, BgGT1 and BgGT3) by immunoblotting. The strains expressing the His-tagged UGTs were cultivated and lysed. After having determined the protein concentration in the soluble fractions, 50 μg of total protein was loaded on SDS-PAGE (Supplementary File 1, Fig. A3d and Table A2) to allow for semi-quantitative comparison of UGT expression in the immunoblot (Fig. 2c). The pre-runs for the immunoblot and the results of the protein quantification are shown in Supplementary File 1, Fig. A3. The immunoblot confirmed the expression of all the UGTs in the yeast strains, but the amount of enzyme produced varied drastically between the strains. DbB5GT was expressed highest, followed by BgGT3 and BvGT2, while CqGT2 and MjcDOPA5GT expression was much lower. SoGT2, which was included in the subset of analysed non-producing UGTs due to its high sequence identity with BvGT2 (76%) and CqGT2 (82%) were expressed at a very low level.

Based on these results, we assumed that most UGTs, betanin-producing and non-producing, could successfully be expressed in soluble form in the betalain production strains, albeit at very different levels. It seemed that for some non-producing UGTs, such as BgGT1 and BgGT3, a lack of functionality rather than low expression or solubility was the reason why they could not produce betanin. Others, however, such as SoGT1, SoGT2 or CqGT1, might have been functional but the activity levels were too low to compensate their low expression. The immunoblot showed that among the betanin-producing UGTs, there was no correlation between the expression level and the amount of betanin produced by the enzyme. This is most clearly seen when comparing the low-expressed but high-producing MjcDOPA5GT with the high-expressed but low-producing DbB5GT. This consequently proved that while the different expression levels influence betanin production, the differences between the UGTs mainly had to stem from the differences in their kinetic properties in the yeast. It should be noted that quantitative analysis by immunoblotting has its limitations and while the presented data shows clear trends for the enzymes’ expression, the results should only be interpreted as semi-quantitative [55]. We only analysed the expression of a subset of the UGTs, and we cannot safely state that the not-tested UGTs are also soluble and expressed in the yeast.

After having identified two additional UGTs that allow the production of betanin, we wanted to see if the ratio of betanin production between the UGTs changed under different media conditions. We cultivated the strains expressing DbB5GT, MjcDOPA5GT, BvGT2, CqGT2 or BgGT2 in MM a. without supplementation, b. with 500 mg/L tyrosine c. with 1.6 mM UDP-glucose and d. with 500 mg/L tyrosine and 1.6 mM UDP-glucose in a small-scale cultivation experiment in 96-well plates (Fig. 2d). Cultivation with UDP-glucose resulted in a slightly lower betanin concentration than without supplementation while the addition of 500 mg/L tyrosine led to a considerable increase in betanin titres. A combination of UDP-glucose and tyrosine supplementation had no symbiotic effect on betanin formation. We concluded that in all strains the acceptor molecule, cDOPA or betanidin, and not UDP-glucose was limiting. The effect of tyrosine supplementation differed slightly between the UGTs so that production increased more than sevenfold for DbB5GT, BvGT2 and CqGT2, sixfold for BgGT2 and fourfold for MjcDOPA5GT. This resulted in CqGT2 and BgGT2 performing better than MjcDOPA5GT in the tyrosine-rich media. It should be noted that the betanin concentrations were determined from the absorbance of the supernatant, which was converted into mg/L with a conversion factor. As can be expected, cultivation in 96-well plates resulted in lower betanin titres than cultivation in 24-well plate. Since we were only interested in the comparison between the strains and between the different media supplementations, this was an acceptable trade-off.

### Screening of UGTs in *Yarrowia lipolytica*

In *S. cerevisiae*, we saw that the highest betanin titres could be achieved with BvGT2. We have previously engineered the oleaginous yeast *Y. lipolytica* as host for betanin production, choosing BvGT2 as glycosyltransferase in the betanin pathway [7]. Before metabolic engineering of the strain, the betanin titres achieved with *Y. lipolytica* expressing a DOD, CYP and BvGT2 were slightly higher but comparable to the ones achieved with *S. cerevisiae* (7–10 mg/L). *Y. lipolytica* is well suited for industrial fermentation of betalains as it is Crabtree negative. Furthermore, it has previously been engineered to produce a remarkably high titre of shikimate-derived products, showing the capacity for high flux through the aromatic amino acid biosynthesis pathway, the building blocks for the red beet pigments [56, 57].

**Fig. 3:**
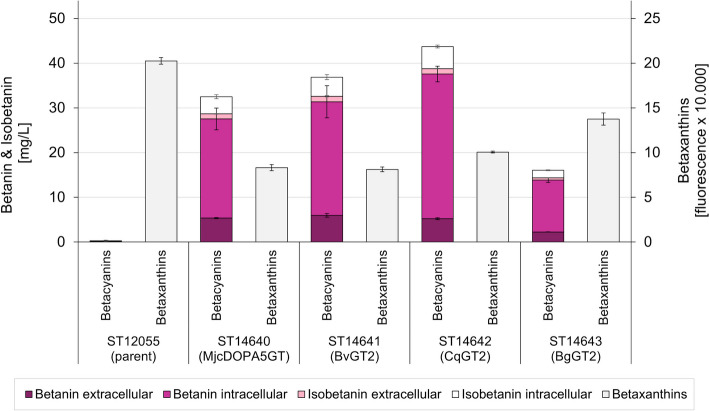
Comparison of betanin production using different UGTs in *Y. lipolytica.* MjcDOPA5GT, BvGT2, CqGT2 and BgGT2 were integrated into the betaxanthin-producing *Y. lipolytica* strain ST12055. The strains were cultivated for 48 h in MM in 24-well plates. Betanin and isobetanin concentrations in the supernatant and total cell extract (supernatant + intracellular) were quantified by HPLC; betaxanthin production of the total cell extract was measured via fluorescence. The mean values of biological triplicates (± SDs) are shown

After screening the GTs in *S. cerevisiae*, we wanted to test CqGT2 and BgGT2 together with BvGT2 and MjcDOPA5GT for betanin production in *Y. lipolytica*. DbB5GT had already been compared to BvGT2 but performed worse and was therefore not included in this experiment. The UGTs, codon-optimised for *Y. lipolytica*, were integrated into strain ST12055, which expressed the betalain pathway genes MjDOD and EvTYH and has been engineered for improved production of L-tyrosine (YlARO4^K221L^ and YlARO7^G141S^). The strains were cultivated in 24-well plates in MM and the (iso-)betanin formation was quantified by HPLC (Fig. 3). Due to the increased tyrosine production, the betanin titres produced by these strains were considerably higher than those produced by the *S. cerevisiae* strains. More than 75% of the betalains were found inside the cells, while in *S. cerevisiae*, almost all betalains were secreted, which is a trend we have observed before [7]. Interestingly, CqGT2, not BvGT2, was the best-performing UGT in *Y. lipolytica* and led to the production of 43.7 mg/L betacyanins in the total cell extract. Apart from that, the order of efficiency of the UGTs to produce betanin stayed the same as in *S. cerevisiae* and was as follows: BgGT2 < MjcDOPA5GT < BvGT2 < CqGT2. The raw data for the cultivated strains can be found in Supplementary File 1, Table A3.

### Structural Analysis and Docking of Substrates to Betanin-Producing UGTs

**Fig. 4:**
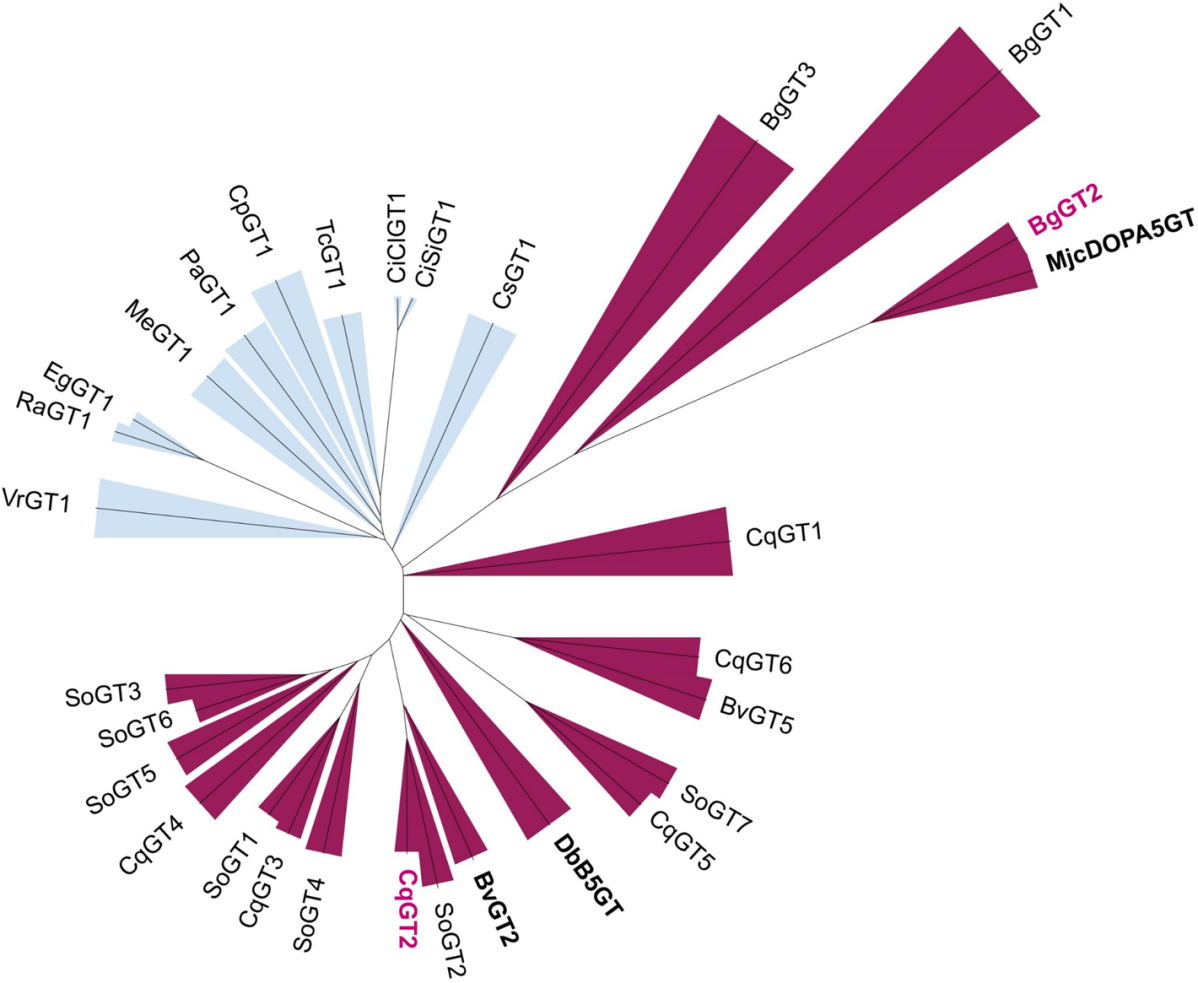
Phylogenetic analysis of 27 UGTs screened for betanin production. UGTs originating from plants from the Caryophyllales order are highlighted red; UGTs originating from plants not producing betalains are highlighted blue. MjcDOPA5GT, DbB5GT and BvGT2 have been shown before to glycosylate the aglycone of betanin, and CqGT2 and BgGT2 (magenta) were identified in this work to make betanin. The phylogenetic tree was constructed using Clustal Omega and iTOL

As mentioned in the “Introduction”, betanin can be produced via two routes. The UGT can take betanidin as an acceptor and attach the glucose at the 5-hydroxyl position of the molecule. Alternatively, cDOPA can be glycosylated, forming cDOPA-5-O-glucoside and afterwards condensate with betalamic acid to betanin. It has been suggested that the UGTs involved in betanin production are selective for their acceptor molecules, and they have therefore been grouped into betanidin-5-O-glycosyltransferases (B5GTs) and cDOPA-5-O-glycosyltransferases (cDOPA5GTs) [17, 31, 58]. According to phylogenetic analysis of all tested UGTs (Fig. 4) and the sequence-based UGT nomenclature (Table 1), BgGT2 groups together with MjcDOPA5GT, while CqGT2 is grouped with BvGT2 and DbB5GT. We have previously performed an in vitro glycosylation assay of BvGT2, which showed that the UGT glycosylates betanidin. Whether BvGT2 can also glycosylate cDOPA has not been assessed [12, 59].

**Fig. 5:**
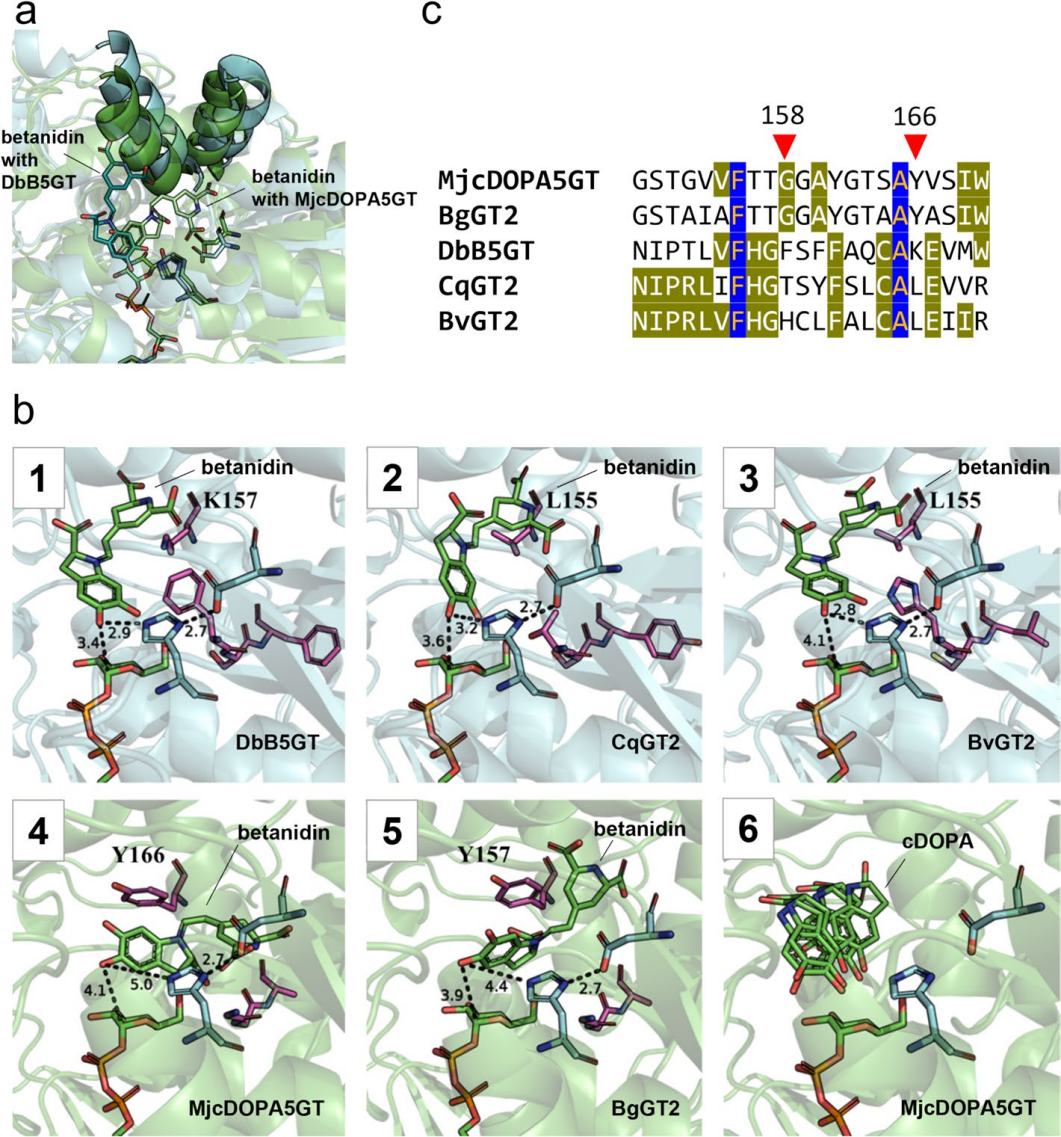
Structural analysis of cDOPA and betanidin glycosylating UGTs. **a** DbB5GT glycosylates exclusively betanidin, MjcDOPA5GT exclusively glycosylates cDOPA. The structural models show a difference between the acceptor binding site size and form between the two UGTs. DbB5GT (cyan) adapts an “outwards-kinked” conformation, while MjcDOPA5GT (green) adapts an “inwards-kinked” conformation. While betanidin fits in the active sites of the “outwards-kinked” enzyme (DbB5GT) without clashes, it adopts a non-reactive pose with MjcDOPA5GT. The catalytic dyad and docked betanidin molecules are colour-coded according to the enzymes. **b** Binding modes of betanidin in DbB5GT (1), CqGT2 (2), BvGT2 (3), MjcDOPA5GT (4) and BgGT2 (5). Binding mode of cDOPA (6), with MjcDOPA5GT in the background. With cDOPA, the dockings for all five enzymes were almost identical. Therefore, the docking with only one protein structure (MjcDOPA5GT) is shown for reference and simplicity. Relevant distances are shown in Å. Catalytic residues are coloured in cyan, other relevant residues in magenta. **c** Part of the global multiple sequence alignment of betanin-producing UGTs that includes the major kinked helix. Positions G158 and Y166 (indexing MjcDOPA5GT) are highlighted as they are suspected to play a role in the different binding mechanisms between the two groups. Amino acids conserved in all the analysed betanin-producing UGTs are highlighted in blue

**Fig. 6:**
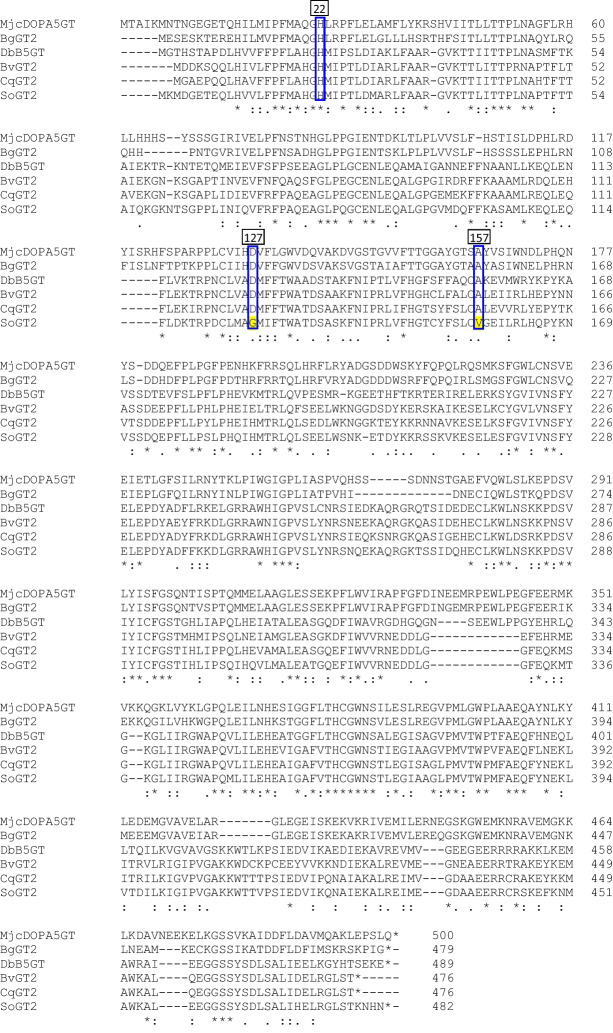
Multiple sequence alignment (MSA) of betanin-producing UGTs and SoGT2.The MSA was made with Clustal Omega (1.2.4). Positions 22, 127 and 157 (indexing SoGT2) are highlighted. D127 and A157 are conserved amongst the tested UGTs that produce betanin but different in SoGT2, whereby position 127 is part of the catalytic dyad of the UGTs. The catalytic dyad consists of position 22 and 127 [24]

To further our understanding of structural determinants of cDOPA and/or betanidin glycosylation by the screened UGTs, we analysed the AlphaFold2 models of the five betanin-producing UGTs: DbB5GT, MjcDOPA5GT, BvGT2, CqGT2 and BgGT2 (bold in Fig. 4). Out of these five, DbB5GT is known to produce betanin primarily via the betanidin glycosylation step (B5GT-clade), while MjcDOPA5GT exclusively utilises cDOPA instead (cDOPA5GT-clade) [17, 58]. While the overall GT-B fold is conserved among the five UGTs, they can be divided into two groups according to their acceptor binding site size and form (Fig. 5a) [60]. Notably, betanidin glycosylating DbB5GT, together with CqGT2 and BvGT2, appeared to feature two N-terminal domain helices in an “outwards-kinked” conformation when compared to MjcDOPA5GT and BgGT2 models, which featured the same helices in an “inwards-kinked” conformation. The “outwards-kinked” helices result in a doubling of the binding pocket volume (1006 Å^3^, 1179 Å^3^ and 1686 Å^3^ for DbB5GT, CqGT2 and BvGT2, respectively, and 563 Å^3^ and 545 Å^3^ for MjcDOPA5GT and BgGT2, respectively). This might explain the capacity of DbB5GT to glycosylate betanidin, while MjcDOPA5GT only glycosylates the smaller cDOPA. To follow up on this, betanidin and cDOPA were docked into the acceptor binding sites of all five UGTs. Interestingly, betanidin only adopted reactive poses with DbB5GT, CqGT2 and BvGT2, which is in accordance with the measured reactivities. While betanidin fits in the active sites of the “outwards-kinked” enzymes (DbB5GT, BvGT2 and CqGT2) without clashes, it adopts a different, non-reactive pose with BgGT2 and MjcDOPA5GT (Fig. 5b). Noticeably, the best betanidin binding modes in MjcDOPA5GT and BgGT2 result in a much lower affinity (29.8 kcal/mol and 26.1 kcal/mol, respectively) than in DbB5GT, CqGT2 and BvGT2 (− 2.8 kcal/mol, − 7.4 kcal/mol, 1.77 kcal/mol, respectively). cDOPA, in contrast, adopts reactive poses in all five enzymes with a similar binding mode and no large differences in calculated affinities. In addition to the observed difference in helix conformation, in MjcDOPA5GT and BgGT2, a deeper binding pocket results from the G158 (MjcDOPA5GT) and G149 (BgGT2) residues, which are bulkier amino acids in DbB5GT, CqGT2 and BvGT2 (F149, T147 and H147, respectively) (Fig. 5c). In the latter group, the binding pocket extends in another direction, parallel to the UDP-glucose binding site and results from the outward kink of the major helix. These two differences allow for the difference in betanidin-binding orientation between “outwards-kinked” and “inwards-kinked” enzymes. Additionally, MjcDOPA5GT and BgGT2 (inwards-kinked) also exhibit Y166, which, together with the protein backbone packing tighter around the binding site, prohibits the binding of betanidin in the same mode as the “outwards-kinked” group. The catalytic dyad of these UGTs consists of H22 and D126 (indexing DbB5GT) (Appendix, Fig. 6) [24]. Interestingly, SoGT2, which groups closely together with DbB5GT and BvGT2 in the phylogenetic tree, is not only missing the conserved aspartic acid (D) in the catalytic dyad (D126 in DbB5GT and G127 in SoGT2) but also has a valine instead of the conserved alanine at position 157 (SoGT2 indexing), corresponding to A165 in MjcDOPA5GT (Appendix, Fig. 6).

The structural analysis and docking models suggest that differences in structure (“outwards-kinked” or “inwards-kinked”) and sequence (positions 158 and 166) between BgGT2 and MjcDOPA5GT on one site and BvGT2, CqGT2 and DbB5GT on the other site influence the acceptor binding sites, altering the acceptor affinity of the enzymes and determining if they glycosylate cDOPA or betanidin.

## Discussion

The success of fermentation-based production depends on the efficiency of the enzymes involved in the biosynthesis pathway. When using heterologous enzymes, choosing efficient enzyme variants is therefore crucial. Three heterologous enzymes are required to produce the red pigment betanin in yeast, the last being a UDP-glycosyltransferase (UGT). The UGT stabilises the pigment’s chromophore and thus allows it to be used as a food colourant.

The first UGTs involved in betanin synthesis were characterised many years ago [17, 18]. But in recent years, multiple studies on betanin-producing plants such as dragon fruit (*Hylocereus* spp.), quinoa (*Chenopodium quinoa*), amaranth (*Amaranthus* spp.) or four-o’clock flower (*Mirabilis jalapa*), often driven by transcriptome analysis, led to the discovery of more UGTs involved in the synthesis of betanin [30, 31, 61–63]. In contrast to these approaches, we used data already available in public databases (NCBI) to find UGTs that efficiently glycosylate the betanin precursors but have not yet been associated with betanin biosynthesis. We selected the best hits from plants producing betalains and plants not producing betalains and tested them by in vivo screening in *S. cerevisiae*. The advantage of this approach is that it allows to screen for those UGTs that are most active in the heterologous yeast system, which is of more relevance to us than the enzyme’s expression and activity in the plant or in vitro. Out of the 27 tested UGTs, only two were able to produce betanin, even though most of the UGTs belonged to the same UGT family (UGT73). For the UGTs from plants that do not make betalains in nature, this is probably due to the enzymes being involved in the glycosylation of other flavonoids (e.g. in *Citrus* spp. or *Prunus avium*) and not being promiscuous enough also to glycosylate betanidin or cDOPA [64, 65]. The low number of betanin-producing enzymes from quinoa and spinach initially surprised us. It can, however, be explained when considering that no expression data was available for the enzyme selection to check whether the selected genes are co-expressed with betalain pathway genes. Some of these UGTs might have taken over different functions in the plants or have lost their function throughout evolution. It is also possible that some of the UGTs are active in the plant but not in the yeast. The abundance of betalain pathway genes has been observed before, for instance, in *Hylocereus* spp., *C. quinoa* and *B. vulgaris* [12, 31, 66]. Transcriptomic analyses in the first two showed that the identified UGTs can have different expression patterns or are expressed in different parts of the plant, suggesting they evolved to play different roles in the plants.

The two new UGTs that enabled the production of betanin in *S. cerevisiae* were CqGT2 and BgGT2, originating from *Chenopodium quinoa* and *Bougainvillea glabra*, respectively. Neither CqGT2 nor BgGT2 have been shown to be involved in betalain biosynthesis before, even though the enzymes involved in betanin synthesis in quinoa have already been investigated [30]. The enzymes’ high activities in the yeast give a strong indication that both play a key role in betanin synthesis in their respective plants of origin, but transcriptomic analysis is needed for confirmation. It is noteworthy that of all tested UGTs, CqGT2 and BgGT2 have the highest sequence identity to BvGT2 and MjcDOPA5GT, respectively, (CqGT2 to BvGT2, 78.4%; BgGT2 to MjcDOPA5GT, 76.6%). Both CqGT2 and BgGT2 produced more betanin in *S. cerevisiae* than the previously known DbB5GT (Fig. 2b), which is particularly interesting when looking at the expression levels of the different UGTs (Fig. 2c). As far as it can be concluded from the immunoblotting, DbB5GT is expressed highest, while BgGT2 and BvGT2 are expressed at lower levels and MjcDOPA5GT and CqGT2 even less. This leads to the conclusion that the activity of BvGT2, MjcDOPA5GT and CqGT2 must be considerably higher than that of DbB5GT to compensate for the lower expression level in the yeast. This not only emphasises the importance of screening enzymes for heterologous production in the appropriate system but also reveals that enhancing the expression of the low-expressed UGTs might be a promising strain engineering strategy. We did not investigate the reasons for the observed difference in expression levels of the UGTs further, but typically, differences in a protein’s solubility and stability have a strong influence on protein expression. When the soluble and insoluble protein fractions were tested, however, almost no UGTs could be detected in the insoluble fractions (Supplementary File 1, Fig. A3) [67]. Transcription and translation efficiency can also differ between the proteins, depending on their codons or N- and C-termini, and thereby influence the individual expression. The fusion-tag can hereby also affect gene expression and cellular processing [68–70]. Furthermore, a difference in the binding efficiency of the antibody to the His-tag or other irregularities during one of the many steps in immunoblotting can impact the detected amount of protein.

We saw that upon supplementation with tyrosine, all strains produced more betanin, clearly showing that substrate is limiting and that strain engineering should aim towards increased tyrosine supply. CqGT2 and BgGT2 performed better than MjcDOPA5GT when tyrosine was fed, while the increase in betanin production was lower for BgGT2 and MjcDOPA5GT. Differences in the enzymes’ kinetics are likely the reasons for this, though we can only speculate about the underlying mechanisms. One possible explanation is that MjcDOPA5GT has a higher affinity towards the acceptor molecule than BvGT2 and CqGT2 (lower *K*_m_), which becomes irrelevant when the substrate concentration is high, while BvGT2 and CqGT2, in contrast, have higher *k*_cat_ values. However, other reasons are also conceivable and without further experiments, no final explanation for the observed trend can be given. Nevertheless, this data showed that the performance of the enzymes is highly dependent on the precursor supply and that their activities should ideally be evaluated in the context of the metabolically engineered production organism.

The betanin titres shown here for *S. cerevisiae* are low and far away from being useful for industrial production of betanin as an alternative to extracted betanin from plants. We could recently demonstrate, however, that this process can successfully be scaled up to betanin titres > 1 g/L by metabolic engineering of the oleaginous yeast *Y. lipolytica* [7]. This is the highest titre of betanin produced in a microorganism reported to date. In the best-producing strain, three copies of BvGT2 were integrated. When testing the other UGTs in a *Y. lipolytica* strain with increased precursor supply, we discovered that in this strain background, CqGT2, not BvGT2, led to the highest betanin titre (43 mg/L). We assume that the different strain background, e.g. different concentrations of UDP-glucose and acceptor molecule or a difference in the expression levels, are responsible for the different activities of the UGTs in *Y. lipolytica* compared to *S. cerevisiae*. For future engineering of *Y. lipolytica* for betanin production, choosing CqGT2 should be considered. However, apart from mere overexpression of the enzymes in a biosynthesis pathway, fine-tuning the metabolic flux can be crucial to achieve high titres. The different activities and mechanisms of the described UGTs could be put to use by combinatorial expression of the UGTs to find the ideal balance of glycosylation, precursor supply and metabolic burden.

Heuer et al. and Sasaki et al. performed in vitro assays, showing exclusive glycosylation of betanidin for DbB5GT and cDOPA for MjcDOPA5GT [17, 58]. Based on those results, UGTs from betanin-producing plants have been grouped into cDOPA5GTs or B5GTs, depending on their sequence and grouping with either DbB5GT or MjcDOPA5GT in phylogenetic analysis [31, 62, 63]. However, the acceptor preference has not been characterised experimentally for any of the newly identified UGTs involved in betalain biosynthesis. We performed structure analysis and docking to elucidate the molecular mechanism behind the reported acceptor specificities of the UGTs. This showed that the acceptor binding site of the cDOPA5GT-clade comprising BgGT2 and MjcDOPA5GT only accommodated cDOPA in an active pose, while the larger site of the B5GT-clade, including BvGT2, CqGT2 and DbB5GT accommodated both betanidin and cDOPA in a catalytically competent pose. To verify the findings from the modelling experimentally, we wanted to perform in vitro glycosylation assays, using either betanidin or cDOPA as acceptor molecules for the purified UGTs as previously reported [17, 58]. Unfortunately, we failed to obtain enough purified enzyme. We, therefore, attempted to use crude yeast extracts of strains expressing only the UGT of interest. However, due to low enzyme concentrations and low substrate concentrations, we could not conclude the acceptor preference of the enzymes. Additionally, the low concentrations increased the chance of false positives due to the spontaneous degradation of betanidin into betalamic acid and cDOPA during incubation, which made it difficult to be sure that betanidin and not cDOPA was glycosylated by the UGTs. Without the experimental validation, while promising, the presented in-silico results should be considered tentative.

The docking suggested that the amino acids in the acceptor binding pocket, particularly at positions 158 and 166, indexing MjcDOPA5GT, determine whether the enzyme can bind cDOPA, betanidin or both. It would be interesting to see whether replacing G158 and Y166, present in BgGT2 and MjcDOPA5GT, with the amino acids found at those positions in the B5GT-clade (F, T or H for position 158 and K or L for position 166) changes the acceptor preference of the UGTs and vice versa.

cDOPA and betanidin are highly unstable molecules. Without stabilisation, more than half of cDOPA degrades within 90 min when incubated at pH > 5.7 and 30 **°**C, while most betanidin will hydrolysate after 30 min [58, 71]. Stabilising both molecules, cDOPA and betanidin, by glycosylation should benefit the heterologous production of betanin as it prevents the loss of valuable carbon to the degradation of either precursor [72]. Therefore, integrating a B5GT and a cDOPA5GT in the production strain should be considered. Fast glycosylation of the valuable precursors would not only prevent their degradation but also allow betanin formation via both glycosylation routes, increasing the flux towards betanin and resulting in higher betanin titres and yields. It can be suspected that it is for the same reason that some betalain-producing plants have both types of UGT, cDOPA5GT and B5GT, e.g. AmB5-GT and AmcDOPA5-GT in *Amaranthus tricolor* [73], HucDOPA5GT1, HucDOPA5GT2 and HuB5GT1, HuB5GT2 in *Hylocereus undatus* [31] or CqCDOPA5GT and the here described CqGT2 in *Chenopodium quinoa* [30]. Furthermore, our results show that in contrast to previous suggestions, using a cDOPA5GT in a heterologous system is not always more efficient but depends on the UGT, its kinetic parameters and the strain background.

## Conclusion

We identified two novel UDP-glycosyltransferases that catalyse the formation of betanin in a heterologous production system of which one, CqGT2, was the best-performing UGT for betanin biosynthesis in *Y. lipolytica*. To our knowledge, CqGT2 and BgGT2 have not been described before and can be expected to play key roles in betanin biosynthesis in *C.quinoa *and *B. glabra*. Furthermore, we could show the importance of in vivo screening of heterologous enzymes when constructing production strains. In silico structural analysis and docking of substrates revealed the potential molecular mechanism underlying the distinct acceptor preferences between the B5GT-clade and the cDOPA5GT-clade on a structural level.

## Electronic supplementary material

Below is the link to the electronic supplementary material.

Supplementary file1 (DOCX 1.27 MB)

Supplementary file2 (XLSX 66.7 KB)

Supplementary file3 (XLSX 16 KB)

## Data Availability

The authors declare that the data supporting the findings of this study are available within the paper and its Supplementary Information files. Should any additional raw data files or biological material be needed, please contact the corresponding author.

## Appendix

See Fig. 6.

## Abbreviations

UGT: UDP-glycosyltransferase
cDOPA5GT: cDOPA-5-O-glycosyltransferase
B5GT: Betanidin-5-O-glycosyltransferase
cDOPA: cyclo-DOPA/ Leucodopachrome
MM: Minimal media
MSA: Multiple sequence alignment
pABA: p-Aminobenzoic acid
L-DOPA: 3,4-Dihydroxy-L-phenylalanine
DOD: DOPA-4,5-extradiol dioxygenase
DTT: Dithiothreitol
SC: Synthetic complete (media)
YPD: Yeast peptone dextrose (media)
