# Supplementary material for: Screening of Plant UDP-Glycosyltransferases for Betanin Production in Yeast
Source: Appl Biochem Biotechnol. 2025 Jan 2;197(4):2356–82. doi: 10.1007/s12010-024-05100-4 (PMC11985647; doi:10.1007/s12010-024-05100-4)
Supplement: Supplementary file 1 — Supplementary file1 (DOCX 1.27 MB) [file 12010_2024_5100_MOESM1_ESM.docx]

**Screening of Plant Glycosyltransferases for Betanin Production in Yeast**

*Christiane Glitz ^a^, Jane Dannow Dyekjær ^a^, Dovydas Vaitkus ^a^, Mahsa Babaei ^a^, Ditte Hededam Welner^a^, Irina Borodina ^a^**

^a^ The Novo Nordisk Foundation Center for Biosustainability, Technical University of Denmark, Kemitorvet Building 220, DK-2800 Kgs. Lyngby, Denmark

*Corresponding author: Irina Borodina, orcid.org/0000-0002-8452-1393, Email: [irbo@biosustain.dtu.dk](mailto:irbo@biosustain.dtu.dk)

[Supplementary Fig. A1 UV-Vis spectrum (normalised to OD660) of the total extract 2](#_Toc171099900)

[Supplementary Fig. A2 HPLC chromatograms of the betanin-producing yeast strains 3](#_Toc171099901)

[Supplementary Fig. A3 Pre-runs for the expression analysis of the UGTs in](#_Toc171099902) *[S. cerevisiae](#_Toc171099902)* [by immunoblotting 5](#_Toc171099902)

Supplementary Table A1 Betacyanin and betaxanthin production in *S. cerevisiae* strains expressing plant UGTs 4

Supplementary Table A2 Protein concentration of the soluble protein fractions from yeast cultures, determined by BCA protein assay 6

Supplementary Table A3 Betacyanins and betaxanthins production in *Y. lipolytica* strains expressing plant UGTs 7


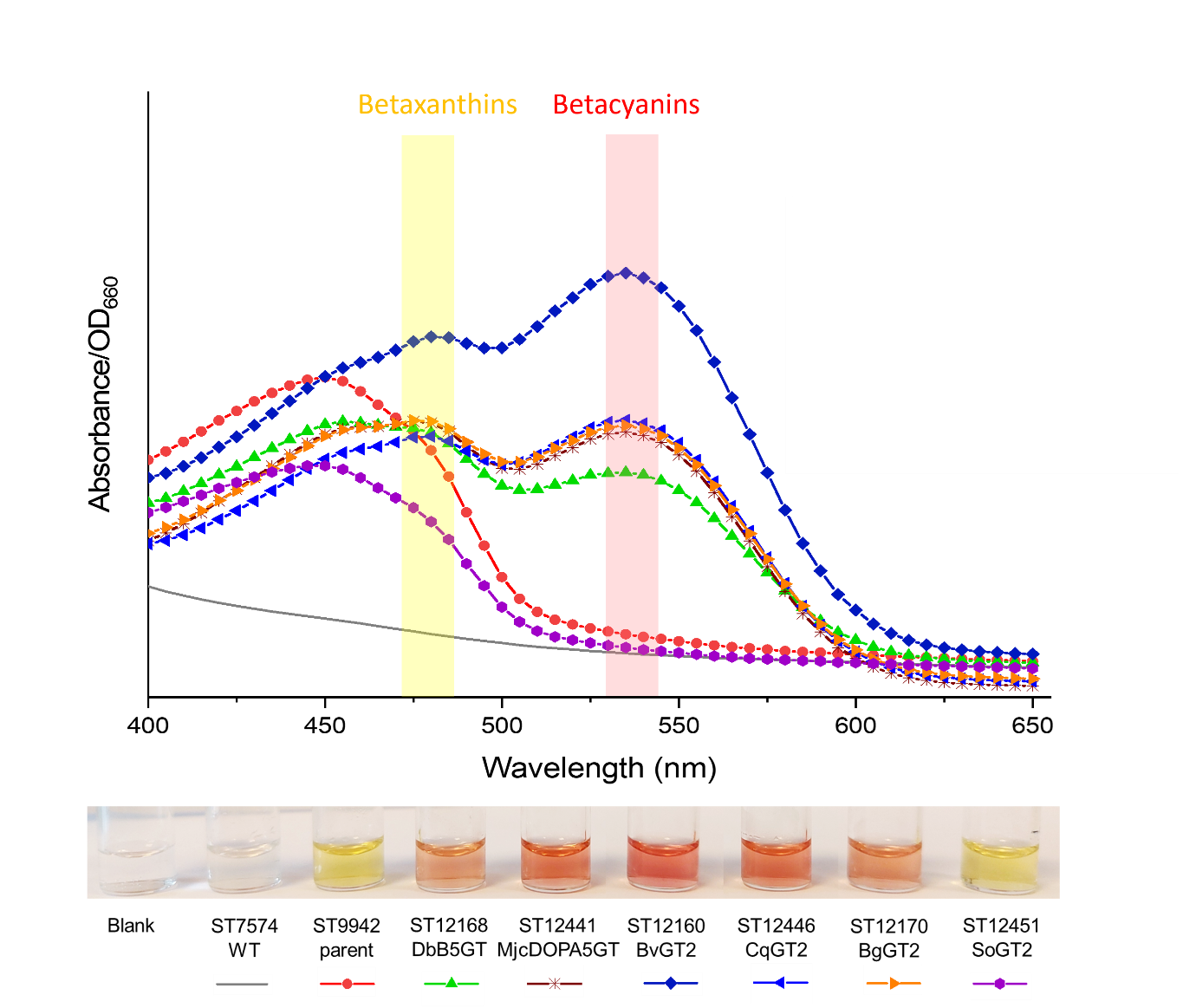


Supplementary Fig. A1

UV-Vis spectrum (normalised to OD_660_) of the total extract. The extracts of strains with integrated DbB5GT, MjcDOPA5GT, BvGT2, CqGT2 and BgGT2 show a red colour and their spectra have a maximum absorbance at ca. 535 nm, the λ_max_ of betanin, and a second peak at ca. 480 nm, the λ_max_ of betaxanthins. The extracts of ST9942 and ST12451 (↑SoGT2) are yellow with max. absorbance at 450 nm

.


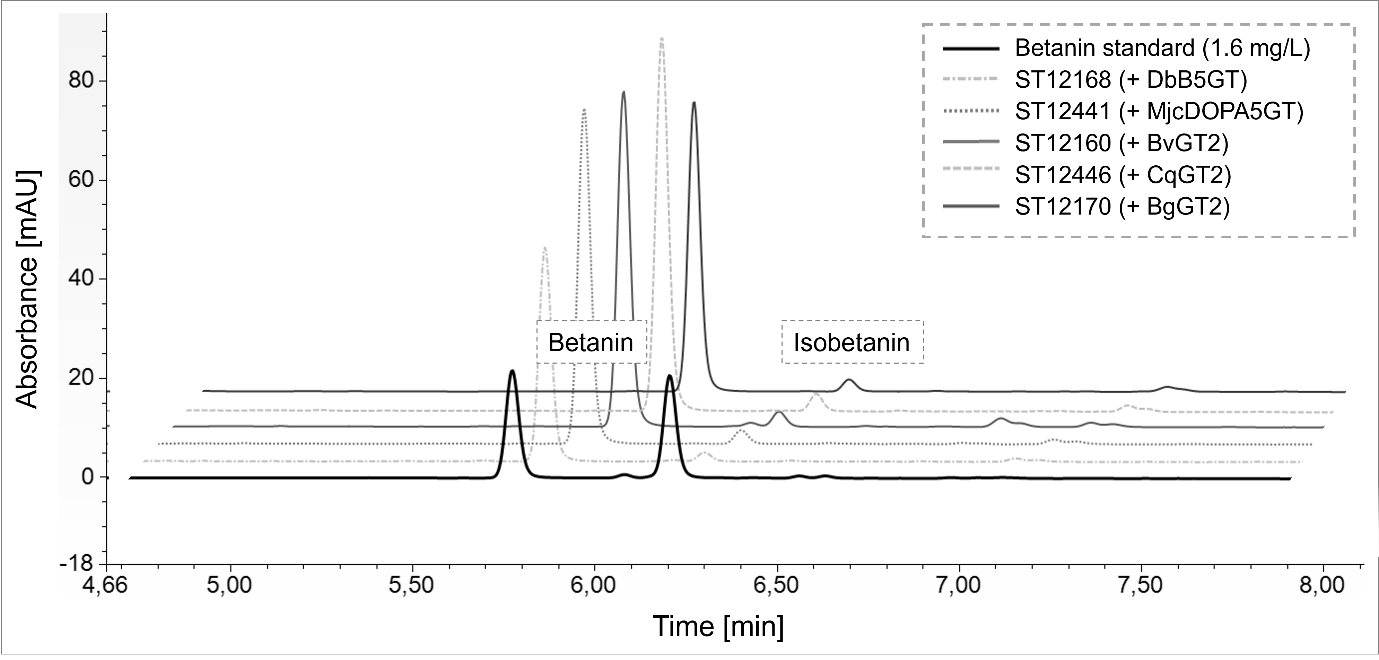


Supplementary Fig. A2

HPLC chromatograms of the betanin-producing yeast strains. The total extracts from the screening of the UGTs in *S. cerevisiae* were analysed by HPLC. The chromatogram at 535 nm is shown. Comparison with a commercial betanin standard allowed the identification of betanin and isobetanin produced by the strains expressing a DbB5GT, MjcDOPA5GT, BvGT2, CqGT2 or BgGT2. The chromatograms are shown with signal and time offset. While the betanin standard contained almost equal amounts of isobetanin and betanin, the fermentation broth contained mainly betanin. In all strains, a very small peak was visible at RT of 7.0 min with a maximal absorbance of 480 nm, likely a betaxanthin. In addition to this, the strain expressing BvGT2 had one additional peak with a RT of 6.8 min, likely corresponding to betanidin. *Bougainvillea* spp. is known to produce 6-O-glycosylated betalains and their production requires a 6-O-glycosyltransferase. No 6-O-glycoside e.g. gomphrenin could be detected in the strain expressing BgGT2, nor in another UGT

Supplementary Table A1

Betacyanin and betaxanthin production in *S. cerevisiae* strains expressing plant UGTs. (Iso-)betanin concentrations were measured by HPLC, betaxanthins were quantified via fluorescence. Blank, ST7574, ST9942, DbB5GT, MjcDOPA5GT, BvGT2, CqGT2 and BgGT2 were cultivated in triplicates. Betanin-producing strains are highlighted in grey

| **Strain** | **Total extract (mg/L)** | **Total extract SD** | **Extracellular (mg/L)** | **Extracellular SD** | **Intracellular (mg/L)** | **Intracellular SD** | **Betaxanthins (fluorescence)** | **Betaxanthins SD** | **OD**  **(mean)** |
| --- | --- | --- | --- | --- | --- | --- | --- | --- | --- |
| blank | 0,00 | 0,00 | 0,00 | 0,00 | 0,00 | 0,00 | - | - | 0,00 |
| ST12160 (BvGT2) | 6,40 | 0,49 | 6,02 | 0,20 | 0,38 | 0,28 | 29794 | 3672 | 12,79 |
| ST12163 (BvGT5) | 0,00 |  | 0,00 |  | 0,00 |  | 40441 |  | 13,15 |
| ST12164 (CqGT1) | 0,05 |  | 0,06 |  | 0,00 |  | 54957 |  | 11,37 |
| ST12165 (PaGT1) | 0,00 |  | 0,00 |  | 0,00 |  | 3159 |  | 13,94 |
| ST12166 (SoGT1) | 0,00 |  | 0,00 |  | 0,00 |  | 47348 |  | 10,46 |
| ST12167 (VrGT1) | 0,00 |  | 0,00 |  | 0,00 |  | 34252 |  | 12,26 |
| ST12168 (DbB5GT) | 2,84 | 0,12 | 2,71 | 0,34 | 0,26 | 0,26 | 34530 | 1285 | 10,77 |
| ST12169 (BgGT1) | 0,00 |  | 0,00 |  | 0,00 |  | 40851 |  | 13,31 |
| ST12170 (BgGT2) | 3,97 | 0,06 | 3,60 | 0,05 | 0,37 | 0,03 | 34668 | 2680 | 10,91 |
| ST12171 (BgGT3) | 0,14 |  | 0,13 |  | 0,01 |  | 55820 |  | 12,30 |
| ST12441 (MjcDOPA5GT) | 4,67 | 0,10 | 5,35 | 0,79 | 0,17 | 0,23 | 37124 | 1382 | 12,33 |
| ST12446 (CqGT2) | 5,32 | 0,07 | 4,99 | 0,09 | 0,36 | 0,04 | 39445 | 3247 | 14,00 |
| ST12447 (CqGT3) | 0,19 |  | 0,15 |  | 0,04 |  | 51707 |  | 10,19 |
| ST12448 (CqGT4) | 0,06 |  | 0,11 |  | 0,00 |  | 57428 |  | 14,66 |
| ST12449 (CqGT5) | 0,08 |  | 0,10 |  | 0,00 |  | 68844 |  | 12,70 |
| ST12450 (CqGT6) | 0,04 |  | 0,00 |  | 0,04 |  | 42272 |  | 11,93 |
| ST12451 (SoGT2) | 0,00 |  | 0,00 |  | 0,00 |  | 40991 |  | 12,97 |
| ST12452 (SoGT3) | 0,05 |  | 0,05 |  | 0,00 |  | 63870 |  | 7,12 |
| ST12453 (SoGT4) | 0,07 |  | 0,09 |  | 0,00 |  | 57991 |  | 14,47 |
| ST12454 (SoGT5) | 0,00 |  | 0,00 |  | 0,00 |  | 67096 |  | 12,60 |
| ST12455 (SoGT6) | 0,04 |  | 0,04 |  | 0,00 |  | 39849 |  | 11,65 |
| ST12456 (SoGT7) | 0,00 |  | 0,00 |  | 0,00 |  | 55182 |  | 9,53 |
| ST12457 (CsGT1) | 0,11 |  | 0,14 |  | 0,00 |  | 61968 |  | 18,19 |
| ST12458 (CiSiGT1) | 0,05 |  | 0,05 |  | 0,00 |  | 58952 |  | 11,00 |
| ST12459 (CiClGT1) | 0,00 |  | 0,05 |  | 0,00 |  | 55340 |  | 15,05 |
| ST12460 (EgGT1) | 0,00 |  | 0,00 |  | 0,00 |  | 47878 |  | 12,99 |
| ST12461 (CpGT1) | 0,00 |  | 0,00 |  | 0,00 |  | 36921 |  | 13,44 |
| ST12462 (MeGT1) | 0,05 |  | 0,05 |  | 0,00 |  | 61289 |  | 13,29 |
| ST12463 (RaGT1) | 0,00 |  | 0,00 |  | 0,00 |  | 33247 |  | 12,86 |
| ST12464 (TcGT1) | 0,00 |  | 0,00 |  | 0,00 |  | 51213 |  | 13,79 |
| ST7574 (WT) | 0,00 | 0,00 | 0,01 | 0,02 | 0,00 | 0,00 | 0 | 0 | 13,19 |
| ST9942 (parent) | 0,00 | 0,00 | 0,00 | 0,00 | 0,00 | 0,00 | 61940 | 3577 | 11,91 |


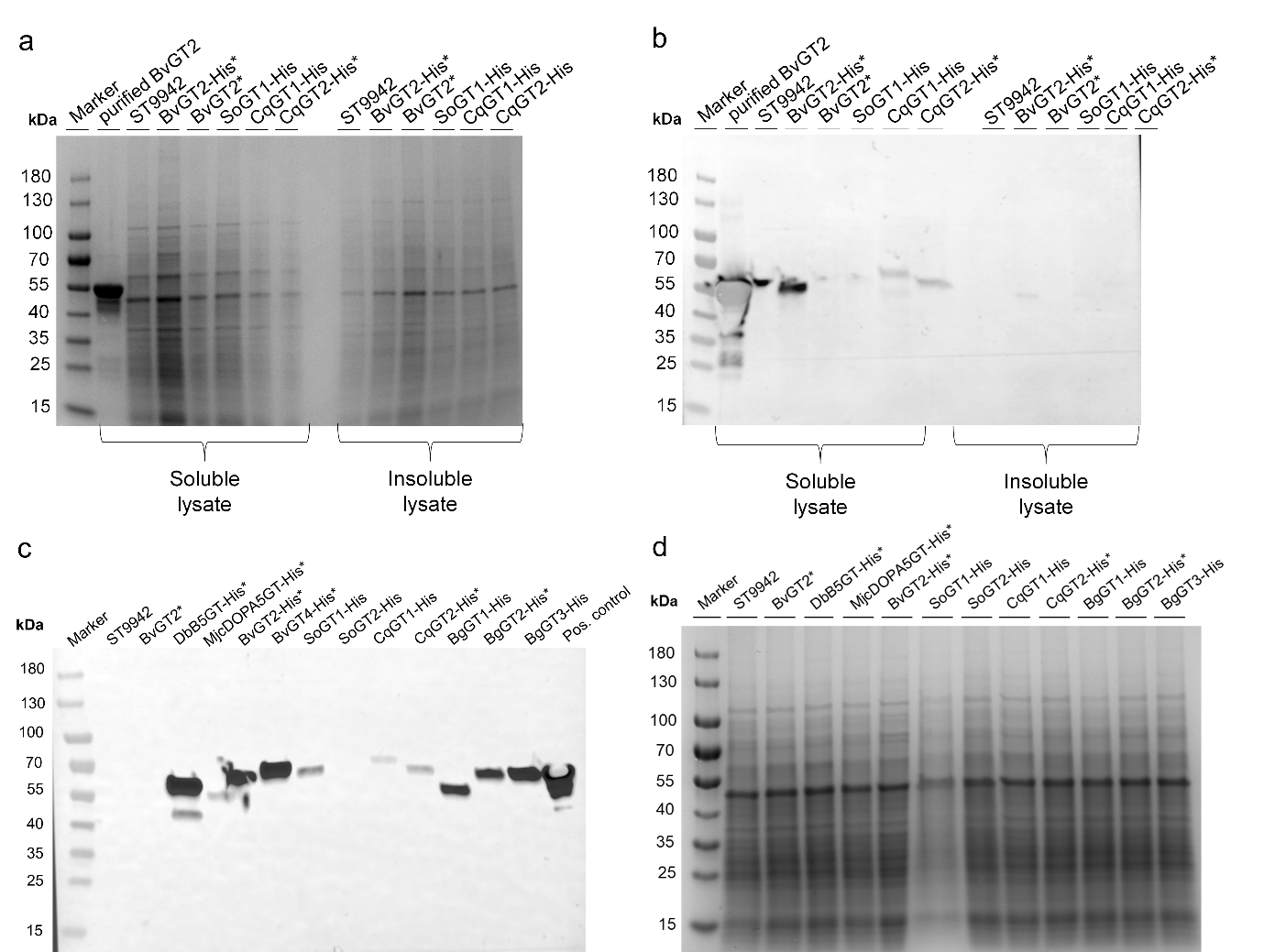


Supplementary Fig. A3

Pre-runs for the expression analysis of the UGTs in *S. cerevisiae* by immunoblotting. All betanin-producing UGTs, marked with an asterix (*), and a subset of non-producing UGTs were fused to a His-tag and integrated into the betaxanthin-producing *S. cerevisiae* strain ST9942. After cultivation of the strains in YPD media, the cells were lysed, and the protein concentrations determined by BCA. (a) Soluble and insoluble cell lysates of some of the analysed strains were separated by SDS-PAGE and stained with Coomassie brilliant blue. (b) Immunoblotting of the lysates in A to verify that the UGTs are present in the soluble fraction. BvGT2-His had been purified from *E. coli* cells in another experiment and served as positive control. (c) Pre-run for the expression analysis of UGTs in yeast production strains by SDS-PAGE and Western Blot. Here, only DbB5GT showed a weak degradation band, for the other UGTs only one band was detected. (d) Coomassie Brilliant Blue-stained SDS-PAGE with 50 μg total soluble protein in each lane, showing that except for SoGT1-His, the same amount of protein was loaded onto the gel. Negative controls: ST9942 (no UGT), BvGT2 (UGT without His-tag). Size of UGTs: ≈ 55 kDa

Supplementary Table A2

Protein concentration of the soluble protein fractions from yeast cultures, determined by BCA protein assay. All betanin-producing and some not-producing UGTs were fused to a 6xHis-tag and integrated in the *S. cerevisiae* strain ST9942. The strains were cultivated for x hours and the cells lysed. The protein concentration in the soluble fraction was determined and used to load an equimolar amount of protein (10 mg/mL) on the SDS-gels for Coomassie staining and immunoblotting

| **Sample** | **Size of fusion construct (UGT-6xHis)** | **Absorbance** | **Protein concentration (mg/mL)** | **Volume on SDS-gel (μl) for 10 (mg/mL)** |
| --- | --- | --- | --- | --- |
| ST9942 | - | 0,0564 | 14,72 | 6,9 |
| ST12604 (ST9442 + DbB5GT-His) | 56.07 | 0,0339 | 10,22 | 10 |
| ST12441 (ST9942 + MjcDOPA5GT-His | 57.41 | 0,0405 | 11,54 | 8,9 |
| ST12605 (ST9942 + BvGT2-His) | 54.93 | 0,0433 | 12,1 | 8,4 |
| ST12607 (ST9942 + SoGT1-His) | 55.91 | 0,0355 | 10,54 | 9,7 |
| ST12515 (ST9942 + SoGT2-His) | 55.26 | 0,0381 | 11,06 | 9,2 |
| ST12608 (ST9942 + CqGT1-His) | 55.87 | 0,0352 | 10,48 | 9,8 |
| ST12609 (ST9942 + CqGT2-His) | 54.43 | 0,0378 | 11 | 9,3 |
| ST12610 (ST9942 + BgGT1-His) | 50.06 | 0,0346 | 10,36 | 9,9 |
| ST12611 (ST9942 + BgGT2-His) | 55.02 | 0,0397 | 11,38 | 9 |
| ST12612 (ST9942 + BgGT3-His) | 56.53 | 0,0409 | 11,62 | 8,8 |
| ST12160 (9942 + BvGT2) | - | 0,0339 | 10,22 | 10 |
| ST12166 (9942 + SoGT1) | - | 0,0411 | 11,66 | 8,8 |

Supplementary Table A3

Betacyanins and betaxanthins production in *Y. lipolytica* strains expressing plant UGTs. Betacyanin concentrations were measured by HPLC, betaxanthins were quantified via fluorescence. The intracellular concentration was calculated by subtracting the extracellular concentrations from the concentrations in the total cell extract

|  | **Extracellular** | | | | **Intracellular** | | | |  | |
| --- | --- | --- | --- | --- | --- | --- | --- | --- | --- | --- |
| **Strain** | **Betanin (mg/L)** | **Betanin SD** | **Isobetanin (mg/L)** | **Isobetanin SD** | **Betanin (mg/L)** | **Betanin SD** | **Isobetanin (mg/L)** | **Isobetanin SD** | **Betaxanthins (fluorescence)** | **Betaxanthins SD** |
| **ST12055**  **(parent)** | 0.05 | 0.07 | 0.03 | 0.04 | 0.15 | 0.18 | 0.00 | 0.00 | 202568 | 3819 |
| **ST14640 (MjcDOPA5GT)** | 5.36 | 0.10 | 1.12 | 0.07 | 22.18 | 2.43 | 3.84 | 0.47 | 83210 | 3467 |
| **ST14641**  **(BvGT2)** | 5.96 | 0.40 | 1.25 | 0.16 | 25.39 | 3.58 | 4.27 | 0.57 | 81263 | 2567 |
| **ST14642**  **(CqGT2)** | 5.23 | 0.21 | 1.21 | 0.06 | 32.35 | 1.75 | 4.92 | 0.32 | 100575 | 1059 |
| **ST14643**  **(BgGT2)** | 2.26 | 0.03 | 0.48 | 0.04 | 11.62 | 0.54 | 1.69 | 0.10 | 137468 | 6809 |
